# Influence of Ionic Liquids on the Selectivity of Ion Exchange-Based Polymer Membrane Sensing Layers

**DOI:** 10.3390/s16071106

**Published:** 2016-07-16

**Authors:** Lukasz Mendecki, Nicole Callan, Meghan Ahern, Benjamin Schazmann, Aleksandar Radu

**Affiliations:** 1 Lennard-Jones Laboratories, Birchall Centre, Keele University, Keele, Staffordshire ST5 5BG, UK; l.mendecki@keele.ac.uk; 2 School of Chemical & Pharmaceutical Sciences, Dublin Institute of Technology, Kevin Street, Dublin 2, Ireland; c12420312@mydit.ie (N.C.); c12329811@mydit.ie (M.A.); Benjamin.Schazmann@dit.ie (B.S.)

**Keywords:** ion-exchange membrane, ionic liquids, potentiometric sensors, selectivity

## Abstract

The applicability of ion exchange membranes is mainly defined by their permselectivity towards specific ions. For instance, the needed selectivity can be sought by modifying some of the components required for the preparation of such membranes. In this study, a new class of materials –trihexyl(tetradecyl)phosphonium based ionic liquids (ILs) were used to modify the properties of ion exchange membranes. We determined selectivity coefficients for iodide as model ion utilizing six phosphonium-based ILs and compared the selectivity with two classical plasticizers. The dielectric properties of membranes plasticized with ionic liquids and their response characteristics towards ten different anions were investigated using potentiometric and impedance measurements. In this large set of data, deviations of obtained selectivity coefficients from the well-established Hofmeister series were observed on many occasions thus indicating a multitude of applications for these ion-exchanging systems.

## 1. Introduction

Ion exchange-based membranes are typically composed of plasticized polymers with the addition of an ion exchange salt. The latter are typically salts that contain a highly lipophilic ion, which enables exchange of only its hydrophilic counter ion with ions of the same charge from the sample thus rendering the membrane permselective. When there are no other complexes formed, the selectivity of sensing layers is based on the energy transfer of ions from the aqueous phase to the organic membrane [1] and therefore is strongly influenced by both the lipophilicity of a targeted ion and the internal composition of a sensing membrane. In this case the selectivity follows the well-established Hofmeister series (ClO_4_^−^ > SCN^−^ > I^−^ > NO_3_^−^ > Br^−^ > Cl^−^ > SO_4_^2−^) [2]. This sequence shows that the most lipophilic anions are preferentially extracted from the sample into the membrane, whereas the transfer of very hydrophilic ions such as sulfates, phosphates and ferrocyanides is energetically less favorable due to their large hydration energy.

Ion exchange-based membranes are utilized in many types of sensors. For example spectroelectrochemical sensors utilize polymeric polyelectrolyte films as the first mode of selectivity that facilitate the selective extraction and pre-concentration of analyte from the sample. This is then followed by ion transport to the optically transparent electrode where the electrochemical and optical signals are measured [3,4,5]. “Electronic tongues” are sensors that utilize a number of low selective membranes in conjunction with advanced mathematical procedures for signal processing and analyte detection [6]. Ion selective electrodes (ISEs), based on the use of a polymeric membrane, rely on the addition of a highly selective ligand typically referred to as an ionophore to facilitate the selective extraction and binding of target analyte [7,8]. This approach greatly enhances the selectivity of the membrane for the specific analyte and allows development of sensitive, portable and inexpensive sensors for selective determination of the activity of ions in aqueous solutions [9,10].

The ability to modulate the selectivity of ion-exchange membranes carries the possibility to expand its application range in fields such as aforementioned electronic tongues, spectroelectrochemical sensors and/or ISEs. The modulation of membrane properties (for example polarity) will dictate the selectivity of the membrane, which, therefore, becomes an important aspect of sensor design. A typical strategy for modulating membrane selectivity is the utilization of different materials used either as lipophilic, ion exchange salt or indeed the matrix itself.

Room temperature ionic liquids (RTILs) are receiving ever-increasing attention in many fields of chemistry. The almost non-measurable vapor pressure, low toxicity, high ionic mobility, large electrochemical window and very good electrical conductivity have stimulated their application as solvating media in the field of analytical chemistry [11]. Since ionic liquids can undergo almost unlimited structural variations (multiple cation and anion combinations) [12], their characteristics can be optimized for a specific target application [13]. The interest in RTILs is growing very quickly in separation science, where their unique properties, such as polarity and low volatility, are associated with non-polar and ionic interactions [14]. Moreover, different functional groups are utilized to promote extraction of the target analyte, thus resulting in different chromatographic [15] and sensing [16] performance. As extraction solvents, RTILs, therefore, have excellent potential for utilization in ion exchange-based sensors [17]. However, it must be noted that the role of RTILs in these sensors is not yet fully understood. For example they have been demonstrated to take the role of inert plasticizers and/or non-specific ion exchangers [18,19], and ionophores [20,21]. While the research in elucidating the role of ILs continues we are building on the knowledge especially from the group of Takachi, who showed that changing the lipophilicity of anion component of IL (A^−^) can favor extraction of positively charged ion from aqueous sample (M^+^) into IL while increasing the lipophilicity of cationic component of IL (C^+^) can favour extraction of anion (B^−^) from aqueous sample into IL [22]. This group has utilized this knowledge to demonstrate many nice applications of ILs not least their use in salt bridges [23,24].

The controversial role of ILs in ion exchange, polymer membrane-based membranes puts pressure to perform systematic studies that will lead towards a full understanding of the role of RTILs. The large structural complexity of RTILs provides enormous potential for development of ion exchange-based membranes which can dictate and modulate efficiency of extraction of ions from water into the IL-based phase [25]. This could be quantified by measuring potentiometric selectivity coefficient of IL-based membranes. In this study we use a range of IL-based membranes and determine their potentiometeic selectivity coefficient in order to study the effect of ILs as plasticizers able to fine-tune response of analyte of choice. We use a range of RTILs based on trihexyl(tetradecyl)phosphonium [P_6,6,6,14_]. In industrial applications phosphonium-based ILs offer superior properties over nitrogen-based ones mainly due to availability and cost [26]. For this work it is also relevant that phosphonium-based ILs serve as a suitable solvent/plasticizer for PVC, which is used as a traditional polymer used in ion-exchange membranes [13,27]. Therefore, we utilized such ILs in order to investigate the hypothesis that RTILs can change the selectivity characteristics of sensing membranes if they are utilized as polymeric plasticizers. It is well known that the dielectric constant is one of key solvent properties and that it depends on RTIL’s composition (more specifically its anion) [28]. It has also been reported that the extraction of hydrophilic anions from the sample into the ion exchange-based membrane may be facilitated if the difference in the dielectric constant (ε) between the sample water and the membrane phase is reduced [1]. As the plasticizer usually comprises over fifty percent of the polymeric membrane, its physicochemical properties would dictate the dielectric constant of the ion exchanger-based membrane and consequently influence its selectivity. Impedance spectroscopy has shown some interesting results in studying the behavior of ionic liquid-polymer inclusion membranes in microbial fuel cells and is therefore used here as well [29].

Herein, we have determined the potentiometric response and calculated potentiometric selectivity coefficients for a range of anions using ionophore-free polymeric membranes plasticized with ILs. We have compared these selectivity coefficients with the ones obtained with membranes using traditional solvent mediators and/or their mixtures with ILs. Selectivity coefficients were evaluated for the determination of the iodide ion, which has been selected as a model ion in this study, in part due to its biological importance [30].

## 2. Experimental Section

### 2.1. Materials

Bis(2-ethylhexyl) sebacate (DOS) (Fluka), 2-nitrophenyl octyl ether (NPOE) (Fluka), poly(vinyl chloride) (Aldrich), tetradodecylammonium chloride (TDAMCl) (Aldrich) were used for fabrication of ionophore free membranes. Ionic liquids, such as trihexyl(tetradecyl)phosphoniumdicyanamide [P_6,6,6,14_][DCA], trihexyl(tetradecyl)phosphoniumbis(trifluoromethanesulfonyl) [P_6,6,6,14_][TFMS], trihexyl(tetradecyl)phosphonium chloride [P_6,6,6,14_][Cl], trihexyl(tetradecyl)phosphoniumdodecylbenzenesulfonate [P_6,6,6,14_][DBS],trihexyl(tetradecyl)phosphoniummethanesulfonate [P_6,6,6,14_][MS], were purchased from Strem Chemicals and had purities >95%. The [P_6,6,6,14_][MO], trihexyl(tetradecyl)phosphoniummethylorange was generously donated by the group of Prof Diamond (Dublin City University, Dublin, Ireland). Structures of all used ILs are provided in Supplemental Info, Figure S1. Solutions of metal ions were prepared in ultra-pure water obtained with a Pico Pure 3 water system. Working solutions of different activities were prepared by serial dilutions of a 0.1 M stock solution.

### 2.2. Preparation of Ionophore-Free Sensing Membranes

Ionophore-free membranes were prepared by dissolving TDAMCl (1 wt %), PVC (33 wt %) and desired plasticizer or RTIL in 0.5 mL of THF. For measurements using electrochemical impedance spectroscopy the aliquot was drop cast onto the top of solid contact electrodes (gold contact) and left at room temperature to dry overnight. The following day, the ISEs were conditioned in a 0.1 M solution of CaCl_2_ for 24 h. For potentiometric measurements, a solution of POT (10^−3^ M of monomer in chloroform) was drop cast onto the top of solid contact electrodes (gold contact) and left at room temperature to dry. This was followed by drop casting the aliquot of membrane cocktail, which was then left at room temperature to dry overnight.

### 2.3. EMF Measurements

Potentiometric responses were recorded with a Lawson Labs Inc. 16-channel EMF-16 interface (3217 Phoenixville Pike, Malvern, PA, USA) in a stirred solution against a double-junction Ag|AgCl|sat. KCl|1M LiOAc reference electrode (Fluka).

### 2.4. Electrochemical Impedance Spectroscopy

Impedance measurements were performed by using a Ivium Technologies CompactStat Impedance Analyzer coupled to a Himux XR 8 channel electrochemical multiplexer (Ivium Technologies, Eindhoven, The Netherlands). The Electrochemical Impedance Spectroscopy (EIS) measurements were performed as described earlier [31]. Briefly, EIS spectra were collected using amplitude of 0.1 V and a frequency range of 100 kHz to 25 Hz. A conventional three electrodes set-up was used in this study using platinum auxiliary electrode and a silver-silver chloride electrode as the reference). Each measurement was carried out at open-circuit potential in 0.1 M solutions of metal salts at room temperature. All impedance spectra were fitted to equivalent circuits using the IviumStat software version 2.0 (Ivium Technologies, Eindhoven, The Netherlands). All measurements were done in at least triplicate. This allowed determination of resistance components and capacitance. The latter was relevant for further calculations. Dielectric constants were calculated, as proposed by O’Rourke [32], using the following equation:

(1) $$C=\;\frac{\varepsilon_{0}\varepsilon_{r}A}{d}$$

where *C* is a capacitance [F], ε_*r*_ is the relative electrical permittivity of the membrane, ε_0_ is the relative permittivity of free space (8.854 × 10^−12^ F·m^−1^), d is the membrane thickness [m] also defined as the distance between two plates of a capacitor and A is the area of the membrane exposed to the aqueous solution [m^2^]. Membrane thicknesses were measured 5 times and then averaged prior to the determination of the dielectric constant.

### 2.5. Selectivity Measurements

Separate solution method: Upon conditioning in 0.1 M CaCl_2_ solution the electrode response was determined for each of the chosen interfering anions separately in the following order: Fe(CN)_6_^4−^, SO_4_^2−^, OH^−^, NO_3_^−^, Br^−^, ClO_4_^−^, SCN^−^, HCrO_4_^−^ and Cl^−^ prior to the determination of the response to iodide.

## 3. Results and Discussion

### 3.1. Role of Ionic Liquids

Prior to embarking on the determination of selectivity coefficients we focused on the determination of the role of ILs in these membranes. In other words, we decided to study whether the IL-based matrix exhibits only-nonspecific interactions with ions of choice, as is the case of traditional plasticizers. We hypothesized that if ILs demonstrate a specific interaction (association) with the ion of choice (in other words behave as ionophore) the occurrence of so called Donna failure should be observed [33].

**Figure 1:**
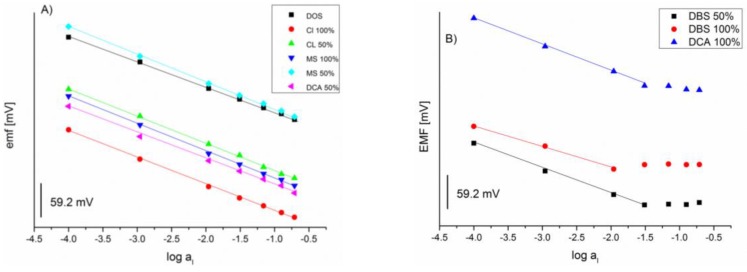
Potentiometric slopes recorded for ion-exchange membranes plasticized with selected RTIL. (**A**) responses of IL^−^ based electrodes that do not show Donnan failure; (**B**) responses of electrodes based on [DCA^−^] and [DBS^−^] exhibiting Donnan failure.

Due to the potentially strong interaction of IL and I− as our ion of choice it’s partitioning into the membrane will be enhanced relative to a simple inert matrix. This would result in the situation where the amount of extracted ion of choice would exceed the one dictated by the amount of ion exchanger thus leading to co-extraction of counterion (K+) in order to preserve electroneutrality of the membrane. Consequentially, deviation from Nernstian response would be observed. In extreme case the response could be completely reversed and electrode would become selective to counterion [34,35]. Observation of Donnan failure can be observed in solutions containing high concentration of ion of choice thus allowing observation of upper detection limit (UDL) [34]. Figure 1 shows responses of IL-based electrodes to iodide. Some deviation from Nernstian slopes is indeed observed but only in the case of membranes ILs based on [DBS−] and [DCA−]. Intuitively, it could be expected that the effect would be reduced with reducing the amount of ILs to 50%. Indeed this is observed in membrane containing [DBS−], while it completely disappears in the membrane based on [DCA−]. Potential reasons for observation of Donnan failure are discussed later in the text when we discuss the dielectric coefficient of [DBS−] based membranes. Overall, we can conclude that almost no studied ILs exhibit specific interaction with ion of choice and are thus suitable for use as plasticizers in ion-exchange membranes.

### 3.2. Selectivity Measurements

**Figure 2:**
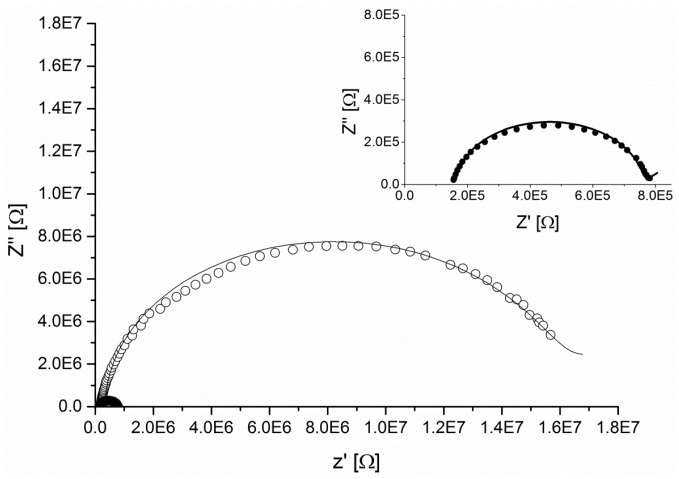
Impedance spectra of the ionophore free membranes plasticized either with DOS (open circles) and [P_6,6,6,14_][DBS^−^] ionic liquid (closed circles). The inset in the upper section of the figure illustrates the Nyquist plot of [DBS^−^] based membranes that is barely visible in the main figure. It can be observed that the presence of ionic liquids decreases membrane’s resistance by the factor of about 20.

Unlike traditional sensing membranes used for decades in potentiometric measurements, ionic liquid based ISEs exhibit significantly different physicochemical properties and exhibit selectivity that not always follows the Hofmeister order. Ionic groups present at high concentrations within the membrane matrix increase the dielectric constant (polarity) of the membrane and subsequently facilitate the extraction of more hydrophilic anions from the sample solution into the organic phase. As traditionally prepared ISEs contain over 50% of a plasticizer, their replacement with a more ionic species significantly reduces the resistance and capacitance in comparison to traditional molecular plasticizers, as demonstrated in Figure 2. The EIS measurements of ion selective membranes can display inductive behavior indicating a resistance to phase change to the frequency scanning of the investigated ISE [32]. If such behavior is observed, the determination of dielectric constant should be avoided as it may introduce a significant bias into the results.

Most of the membranes under study, with the exception of the DBS^−^, DOS and NPOE plasticized ISEs, showed extremely low resistances and capacitances, which could not be accurately quantified. These results imply a very ionic nature of tested membranes and therefore excessively high dielectric values. On the contrary, both membranes containing traditional plasticizers displayed much higher resistance with the NPOE based sensors being more conductive than those plasticized with DOS. This can be explained by the more polar nature of NPOE as evidenced by the values of dielectric constant measured. These findings are summarized in Table 1.

To validate whether the highly ionic nature of the investigated membranes had a direct influence on the selectivity of ISEs, the potentiometric response of each membrane was measured. Three electrodes of each type were prepared and their working characteristics in the separate solutions of various anions (ClO_4_^−^, SCN^−^, NO_3_^−^, I^−^, HCrO_4_^−^, OH^−^, Br^−^, Cl^−^, Fe(CN)_6_^4−^ and SO_4_^2−^) were evaluated. All electrodes responded with theoretical Nernstian or near-Nernstian slopes upon adding known concentrations of interfering anions to the sample solution with ultrapure water as background. Every studied membrane responded rapidly (t_response_ ≤ 7 s) to changes in the concentration of background ions, producing a stable signal. Short-term stabilities were also evaluated from the potentiometric data. The gradual change in the response of tested ISEs (electromotive force (EMF) vs. time) provided information about the electrodes short-term stability, with values not exceeding 0.2 mV·min^−1^. These obtained results are promising for practical applications.

Sensing membranes plasticized with ILs typically exhibited the selectivity that is characteristic to the Hofmeister sequence with its magnitude deviating significantly from the ISEs prepared with traditional plasticizers. It was especially apparent that the most lipophilic membranes (containing DOS) favored the extraction of ClO_4_^−^ over iodide ions by about three orders of magnitude while the same ion was less preferentially extracted when more polar membranes were used—K^POT^ = 2.5 for NPOE and between 1.7 and 0.7 for IL based ISEs. As suggested earlier, the resulting increase in polarity may be accounted to the high content of IL moieties within the membrane segment. Their presence decreases the overall lipophilicity of the membrane and minimizes the energy barrier for more hydrophilic ions to cross the phase boundary interface. These findings are strongly supported by the EIS data presented earlier in Table 1.

Sensing membranes with low dielectric constant values (plasticized with DOS) favored extraction of more lipophilic ions such as perchlorates or thiocyanates, however when the overall hydrophilicity of these membranes was increased (higher ε) the selective response towards these anions approached those of more hydrophilic ions (ε-DOS > NPOE > IL based membranes). The difference in polarity between investigated IL based membranes could not be calculated from the EIS measurements as the majority of these electrodes exhibited similar behavior to pure capacitors or showed an inductive behavior (Figure S2). Even though, the polarity of each membrane could not be quantified, the capacitive behavior indicates extremely polar (ionic) nature of investigated samples as reported by O’Rourke [28] and, therefore, it supports the findings from the potentiometric experiments.

Interestingly, out of tested IL based membranes, only those plasticized with [P_6,6,6,14_][DBS^−^] produced responses that allowed for the quantification of dielectric constant values. Dodecylbenzenesulfonate anions have previously been demonstrated to form micellar aggregates in aqueous solution if the critical micelle concentration (CMC) is reached [32]. It could be hypothesized that DBS^−^ anions at very high concentrations, such as those used during the preparation of ISEs, may form reversed micellar aggregates. This would lead to a decrease in the number of ionic species present in the membrane and subsequently result in larger resistance of the sensing membrane. Interestingly, ion selective membranes plasticized with a 1:1 mixture of DOS and DBS^−^ ionic liquid displayed a significantly higher dielectric constant (Table 2) than membranes containing pure IL as a plasticizer. Since the overall concentration of DBS^−^ anions was reduced, it is possible that the CMC was not reached and therefore what may appear to be counterintuitive, were more ionic species (carrying charge) present in the membrane. With the increasing concentration of DOS (2:1 ratio), the dielectric constant of such membranes was observed to be much lower as the more lipophilic character of that plasticizer would start dominating and defining membrane characteristics. This is illustrated in Table 2. Further studies on the formation of IL aggregates in ion sensing membranes are in progress in our laboratory.

**Figure 3:**
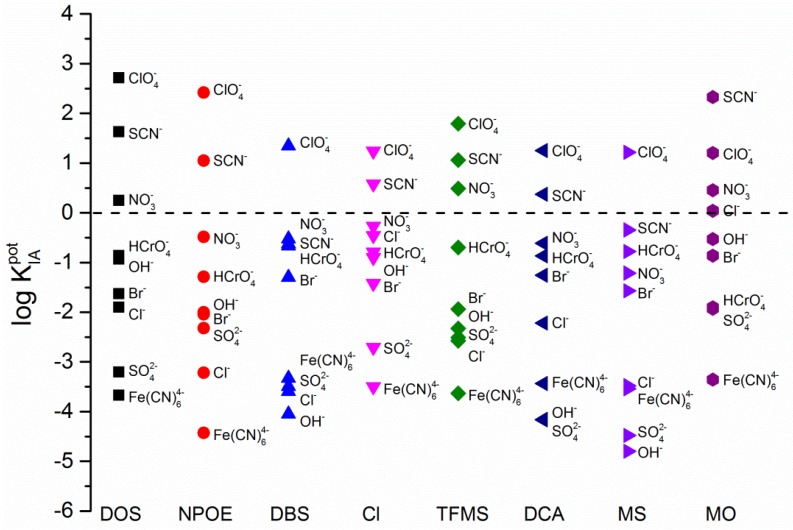
Logarithmic value of selectivity coefficients of iodide (I^−^) versus noted interfering ion (A^−^) obtained by using the separate solution method and utilizing noted plasticizer as solvent mediators. The dotted line indicates log K_POTI,I_ thus equaling zero.

Further potentiometric experiments revealed that the majority of IL based ISEs favored extraction of ions with small hydration radii as predicted by the ion-exchange theory. The perchlorate and thiocyanate anions were favored over better-hydrated ions such as bromide or chloride in all IL-based membranes except the ones based on MO. It has been reported that a ClO_4_^−^ ion is usually preferred by approximately 4.5–5 orders of magnitude over Cl^−^ ions [33], however, much smaller differences (approximately 1 order of magnitude) were observed for membranes plasticized entirely with [P_6,6,6,14_][MO] ionic liquid (Figure 3).

The same membrane demonstrated a very narrow selectivity range (small differences in the selectivity coefficient values) indicating a relatively non-selective nature of this membrane. Such membranes that exhibit a non-selective response have been already indicated as potential candidates for the separation science detectors [34] and for the analysis of abundant hydrophilic anions in the presence of more lipophilic interfering ions. Interestingly, the same membrane started exhibiting a more selective response towards lipophilic ions when the same IL was mixed with DOS at 1:1 ratio (Figure S3). This shows that with an addition of a more lipophilic plasticizer the response characteristics can be reversed providing extra functionality to the membrane.

Table 3 summarizes all numerical values of selectivity coefficients while Figure 3 is its accompanying illustration. From Figure 3 it could be seen that some hydrophilic ions were preferentially extracted into the membrane bulk over the more lipophilic anions depending on the specific IL based membrane. For example, the extraction of chloride anions in [TFMS^−^] and [DBS^−^] based membranes appears to be less energetically favorable than the extraction of sulfate or bromide ions. Whereas, sensing membranes containing [MO^−^] moieties responded preferentially to the nitrate ions rather than ClO_4_^−^ anions. Moreover, most of the ISEs favored extraction of nitrate ions over dichromate ions as predicted by the ion-exchange theory with the exception of membranes containing [MS^−^] species that showed more selective response towards dichromate ions. 

The majority of membranes exhibited a very limited (unselective) response to ferrocyanide anions due to their high hydration energy. However, the ion exchange membranes plasticized with [P_6,6,6,14_][DCA^−^], [DBS^−^] and [MS^−^] favored the diffusion of Fe(CN)_6_^4−^ from the sample into the membrane bulk over the extraction of less hydrophilic sulfate anions. 

On the contrary to [Cl^−^] based ISEs, all [TFMS^−^] based membranes exhibited more selective response towards SO_4_^2−^ and OH^−^ and Br^−^ ions that to the Cl^−^ anions. The enhanced selectivity towards chloride ions was expected for the sensing membranes loaded with Cl^−^ ions prior the measurements as their high concentration dictates the response characteristics of these membranes. Even though, the ion exchange at the sample-membrane interface will result in the presence of other, interfering ions in the membrane, their concentration will be significantly lower than that of the chloride ions. This will either result in the mixed potential response as both interfering and primary (Cl^−^) ions will be present in the membrane bulk and contribute to the phase boundary potential or it will produce a response that is characteristic to the chloride ions only. 

This may imply that IL based membranes containing the same anion as the ion that is being detected have the potential to exhibit high selectivity towards that ion. Furthermore, with the addition of a selective ionophore, sensing properties of these membranes may be improved to produce the characteristic response even in the presence of interfering ions in the investigated sample. The same membrane became less selective towards the chloride ions when half of the [P_6,6,6,14_][Cl^−^] ionic liquid was replaced with DOS (please see Figure S3 and Table S2, and attached discussion). Addition of this traditional plasticizer reduced the total concentration of Cl^−^ ions in the membrane bulk and therefore minimized their contribution towards the overall chloride selectivity. Again, this illustrates that selective response of ISEs can be tuned by preparing mixtures of plasticizers used for membrane preparation [35].

The selectivity of the studied polymeric membranes could be expected to become less dependent on the ion lipophilicity as the high concentration of polar groups (ILs) within the membrane matrix would result in a more pronounced presence of Coulombic interactions between sample ions and charged IL moieties. The presence of more hydrophilic IL groups can also facilitate the diffusion of water from the sample into the membrane bulk affecting the overall polarity of the ISEs and therefore their selectivity [34].

## 4. Conclusions

The selectivity of ion exchange membranes is an important parameter that defines the applicability of these sensors for measurements in both clinical and environmental samples since these specimens often exhibit highly complex characteristics (presence of many interfering ions). Membrane matrices composed of plasticized polymers are one of the key factors that dictate the selectivity of ionophore-free ion exchanger-based sensors. RTILs based on trihexyl(tetradecyl)phosphonium [P_6,6,6,14_] cation have showed almost non-existent specific interaction with ion of choice and are thus useful to serve as plasticizer in ion exchange membranes. Moreover, their capability to influence the extraction of sample ions into the membrane is illustrated by the change of potentiometric selectivity coefficient. The modification was particularly significant for hydrophilic ions since for some ions the selectivity sequence was altered from the traditional Hofmeister series. For example, the most striking example is the change of selectivity of I^−^ vs. Cl^−^ of up to 3 orders of magnitude between DBS- and Cl-based ILs as well as between MS- and MO-based membranes. Similar observations were made for OH^−^ between DCA- and DOS-based membranes. In addition, NO_3_^−^ was preferred to I^−^ in DOS-, TFMS-, and MO-based membranes. These findings are in accordance with higher dielectric constant of RTIL-based membranes. We have also demonstrated that the variations in selectivity can be fine-tuned by the addition of molecular plasticizers to RTIL-based membranes. Overall, we show that RTILs can be successfully utilized as plasticizers in ion exchanger-based membranes, which can be used to finely tune their selectivity.

## Figures and Tables

**Table 1:** Dielectric constants of the membranes plasticized entirely with either traditional plasticizers or ionic liquids obtained from the impedance measurements. Since all IL based membranes contained the same form of a cation—[P_6,6,6,14_], for the simplicity of data presentation, only their corresponding anions are listed.

PVC Membrane Composition	Dielectric Constant
33% PVC, 66% DOS	13 ± 1
33% PVC, 66% NPOE	23.6 ± 0.5
33% PVC, 66% DBS^−^	40.0 ± 0.4
33% PVC, 66% DCA^−^, Cl^−^, TFMS^−^, MS^−^, MO^−^	N/A

**Table 2:** Dielectric constants of [P_6,6,6,14_][DBS^−^] based membranes obtained from EIS measurements.

PVC Membrane Composition	Dielectric Constant
33% PVC, 66% DBS^−^	40 ± 2
33% PVC, 50% DOS and 50% DBS^−^	312 ± 8
33% PVC, 66% DOS and 33% DBS^−^	48 ± 1

**Table 3:** Selectivity coefficients values (log K_POT I-,A-_) calculated for the ion selective membranes that were fully plasticized with either traditional solvent mediators such as DOS and NPOE or [P6,6,6,14] based ionic liquids.

Anion/Type	DOS	NPOE	[DBS^−^]	[Cl^−^]	[TFMS^−^]	[DCA^−^]	[MS^−^]	[MO^−^]
**ClO_4_^−^**	2.71 ± 0.04	2.42 ± 0.11	1.35 ± 0.26	1.24 ± 0.05	1.79 ± 0.03	1.25 ± 0.02	1.22 ± 0.06	1.20 ± 0.11
**SCN^−^**	1.63 ± 0.10	1.05 ± 0.10	−0.52 ± 0.05	0.58 ± 0.09	1.06 ± 0.13	0.37 ± 0.09	−0.35 ± 0.06	2.33 ± 0.04
**NO_3_^−^**	0.25 ± 0.03	−0.49 ± 0.00	−0.62 ± 0.08	−0.27 ± 0.08	0.49 ± 0.03	−0.61 ± 0.09	−1.21 ± 0.07	0.45 ± 0.03
**HCrO_4_^−^**	−0.86 ± 0.16	−1.29 ± 0.13	−0.67 ± 0.06	−0.79 ± 0.07	−0.70 ± 0.05	−0.87 ± 0.01	−0.78 ± 0.15	−1.90 ± 0.06
**OH^−^**	−0.93 ± 0.04	−2.00 ± 0.06	−4.05 ± 0.13	−0.90 ± 0.11	−2.33 ± 0.02	−4.16 ± 0.09	−4.80 ± 0.09	−0.53 ± 013
**Br^−^**	−1.63 ± 0.02	−2.05 ± 0.01	−1.30 ± 0.03	−1.42 ± 0.07	−1.94 ± 0.12	−1.26 ± 0.04	−1.57 ± 0.04	−0.86 ± 0.01
**Cl^−^**	−1.90 ± 0.08	−3.22 ± 0.02	−3.59 ± 0.01	−0.46 ± 0.01	−2.58 ± 0.01	−2.22 ± 0.13	−3.48 ± 0.01	0.04 ± 0.01
**Fe(CN)_6_^4−^**	−3.67 ± 0.06	−4.43 ± 0.01	−3.33 ± 0.06	−3.50 ± 0.13	−3.63 ± 0.05	−3.44 ± 0.11	−3.54 ± 0.06	−3.36 ± 0.10
**SO_4_^2−^**	−3.21 ± 0.01	−2.33 ± 0.00	−3.49 ± 0.12	−2.71 ± 0.01	−2.51 ± 0.03	−4.17 ± 0.01	−4.48 ± 0.01	−1.93 ± 0.02

## Supplementary Materials

Further experimental details. This material is available free of charge via the Internet at www.mdpi.com/1424-8220/16/7/1106/s1.
