# Supplementary material for: Influence of Ionic Liquids on the Selectivity of Ion Exchange-Based Polymer Membrane Sensing Layers
Source: Sensors (Basel). 2016 Jul 16;16(7):1106. doi: 10.3390/s16071106 (PMC4969841; doi:10.3390/s16071106)
Supplement: Supplementary file 1 [file sensors-16-01106-s001.pdf]

Supplementary Materials: Influence of Ionic Liquids on the Selectivity of Ion Exchange-Based Polymer Membrane Sensing Layers

Lukasz Mendecki, Nicole Callan, Meghan Ahern, Benjamin Schazmann and Aleksandar Radu

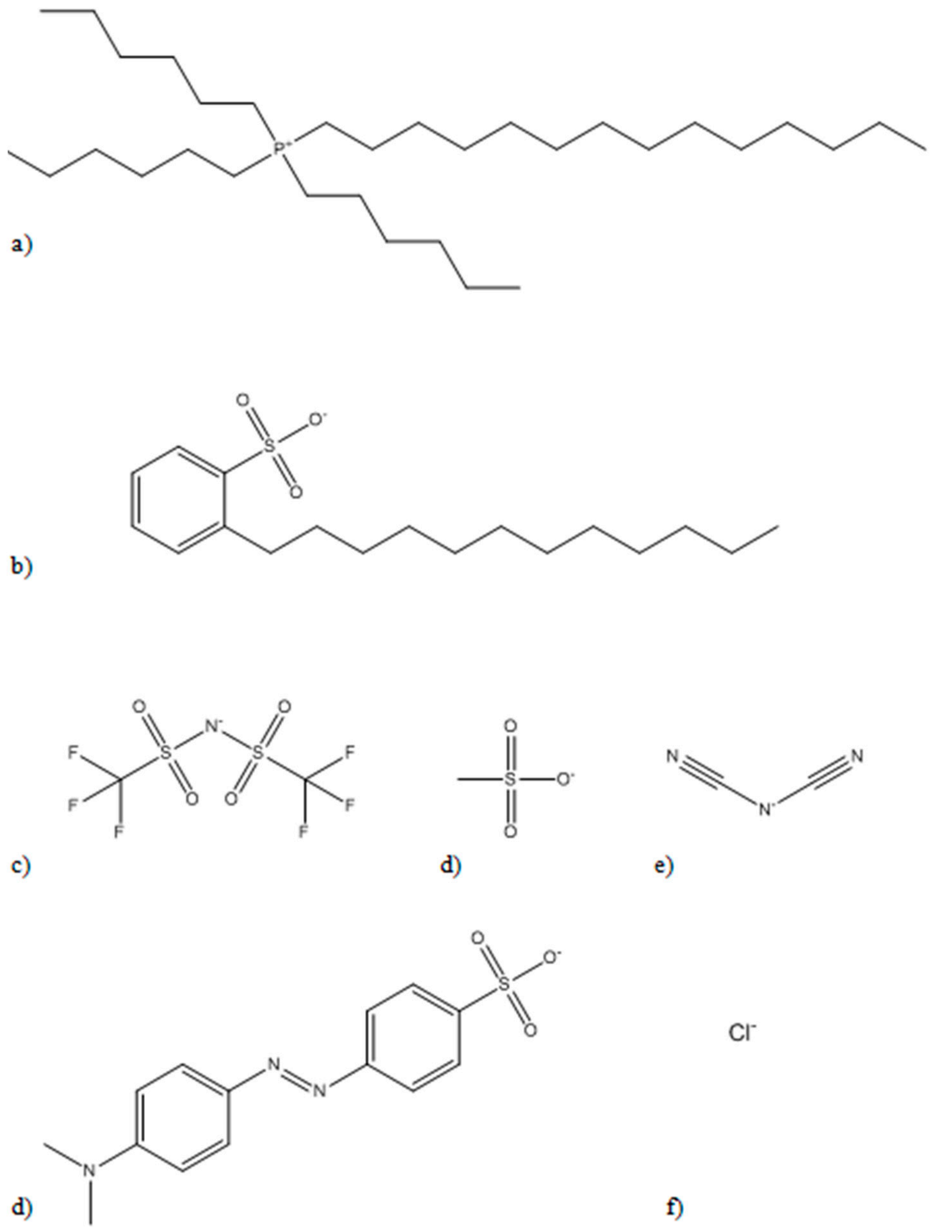

Figure S1. RTILs used for the preparation of ion-exchange membranes; (**a**) trihexyl(tetradecyl)phosphonium cation; (**b**) dodecylbenzenesulfonate anion; (**c**) bis(trifluoromethylsulfonyl)amide anion; (**d**) methanesulfonate anion; (**e**) dicyanamide anion; (**f**) methyl orange anion and g) chloride anion.

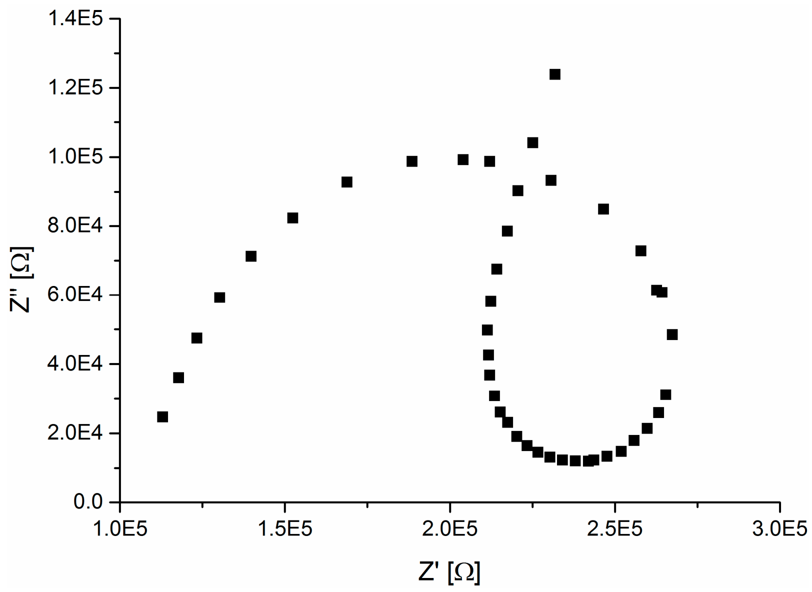

Figure S2. Inductive behaviour of the ion selective membrane plasticised fully with [MS⁻] ionic liquid. Similar inductive responses were observed for the majority of membranes plasticised entirely with ionic liquids. However, the determination of dielectric constants from such membranes was not carried out in order to minimise measurement bias and therefore such values were only calculated from well-defined semicircles as illustrated in Figure 1.

Table S1. Experimental slopes (mV/decade) obtained during potentiometric measurements for ion selective membranes entirely plasticised with ionic liquids or traditional plasticisers.

Anion/Type	DOS	NPOE	DBS	Cl	TFMS	DCA	MS	MO
ClO_4^-	−66.8 ± 3.2	−67.7 ± 2.2	−66.9 ± 4.3	−57.7 ± 0.6	−55.0 ± 6.2	−65.6 ± 1.0	−80.8 ±1.2	−89.6 ± 3.8
SCN^-	−57.5 ± 1.6	−54.4 ± 1.5	−64.6 ± 1.7	−45.9 ± 0.7	−50.8 ± 3.0	−54.1 ± 2.0	−63.1 ± 3.3	−35.1 ± 2.0
I^-	−65.9 ± 2.7	−54.8 ± 1.1	−60.2 ± 5.0	−56.9 ± 5.6	−56.9 ± 5.3	−53.4 ± 5.8	−58.4 ± 5.1	−68.7 ± 1.0
NO_3^-	−65.3 ± 3.2	−56.6 ± 0.6	−56.1 ± 4.3	−49.2 ± 5.8	−62.2 ± 3.0	−52.3 ± 0.0	−48.5 ± 5.6	−51.1 ± 1.2
$HCrO_4^-$	−57.3 ± 4.9	−61.3 ± 2.0	−63.0 ± 2.9	−51.2 ± 0.6	−56.6 ± 3.2	−61.0 ± 3.1	−64.1 ± 3.0	−62.3 ± 4.6
OH^-	−55.4 ± 4.0	−41.7 ± 1.0	−29.2 ± 0.8	−36.8 ± 0.9	−57.1 ± 4.8	−34.9 ± 0.8	−47.4 ± 3.1	−44.9 ± 0.3
Br^-	−53.7 ± 2.1	−56.2 ± 1.8	−54.0 ± 0.7	−44.9 ± 1.8	−61.3 ± 2.3	−47.5 ± 1.6	−59.9 ± 6.0	−45.2 ± 1.4
Cl^-	−56.5 ± 4.0	−56.8 ± 1.9	−57.6 ± 5.5	−61.4 ± 3.1	−61.9 ± 0.0	−55.9 ± 5.9	−58.2 ± 4.5	−59.4 ± 7.3
$Fe(CN)_6^{4-}$	−16.6 ± 1.3	−16.2 ± 1.0	−14.9 ± 0.8	−18.7 ± 3.7	−21.0 ± 1.3	−16.5 ± 0.4	−12.6 ± 3.5	−18.5 ± 1.8
SO_4^{2-}	−20.9 ± 0.6	−25.2 ± 4.4	−32.3 ± 0.3	−37.0 ± 0.7	−34.0 ± 3.3	−33.5 ± 2.0	−33.9 ± 1.7	−31.4 ± 5.9

The majority of tested electrodes produced a Nernstian or near-Nernstian response during the potentiometric measurements with the exception of experiments ran for the determination of perchlorate ions for [MS⁻] and [MO⁻] plasticised membranes. Such super Nernstian behaviour was observed for these two membranes only.

Selectivity of Membranes Plasticised with Mixtures of DOS and IL at 1:1 Ratio

Each of the ionic liquids used in this study was later mixed with a traditional plasticizer—DOS at 1:1 ratio (33% of DOS and 33% IL) to prepare a new set of ion selective membranes. The same protocols were used as described in the experimental section. After conditioning in a 0.1 M solution of calcium chloride overnight the response characteristics of each ion exchange membranes were evaluated using a potentiometric setup. The selectivity coefficient values determined for each membrane are demonstrated in Figure S1 and Table S2.

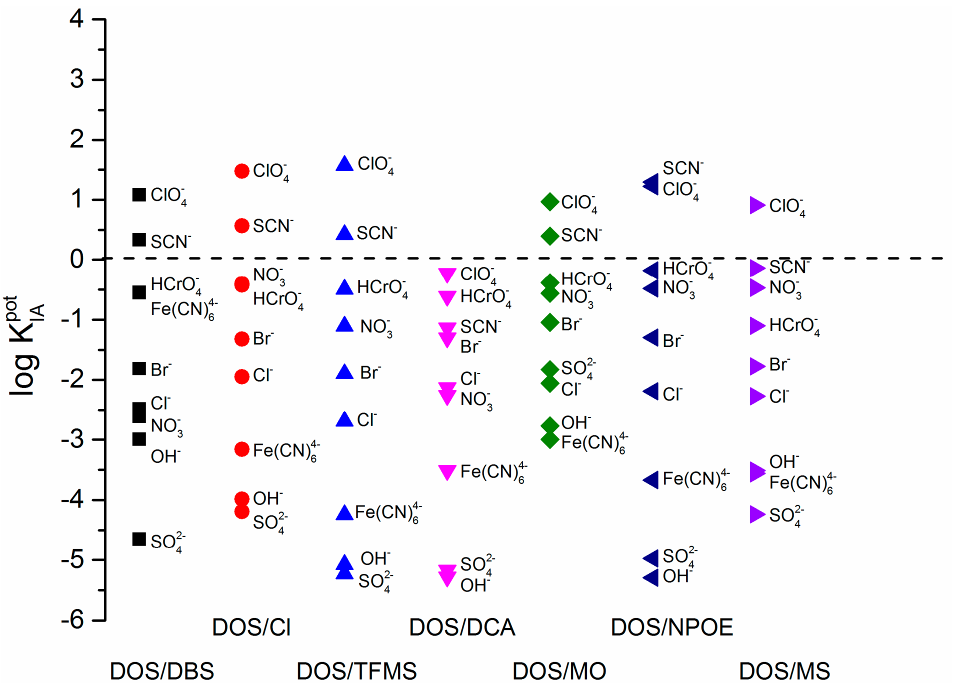

Figure S3. Logharitmic value of selectivity coefficients of iodide (I^-) versus noted interfering ion (A^-) obtained by using the separate solution method and utilizing noted plasticizer as solvent mediators. The dotted line indicates $\log K^{POT}_{I,I}$ thus equalling zero.

On many occasions, similar selectivity patterns were observed for membranes containing mixtures of DOS and ILs at 1:1 ratio as to ion exchange membranes entirely plasticised with ILs or traditional plasticisers. However, some other small deviations in the selectivity pattern could be noticed when a more lipophilic plasticiser was introduced into the membrane bulk. Ion selective membranes plasticised with mixtures of DOS and [DBS^-] or DOS and [$TFMS^-$] started producing responses that reflected the Hofmeister sequence (more lipophilic ions are preferentially extracted into the membrane). This in-part was expected as the overall lipophilicity of the membrane was increased upon the addition of DOS. Similar observations were made for membranes plasticised with [MO^-] ionic liquids where not only the selectivity sequence had changed but also the magnitude of the response was altered. Again, this could be explained by the reduced hydrophilicity of the membrane, which resulted in less preferential extraction of ions such as chloride or hydroxide. This illustrates that the response characteristics of ion selective membranes plasticised with ILs can be partially controlled and modified with the addition of plasticisers of known polarity (such as DOS) and therefore the effects of such combinations can somehow be predicted.

Table S2. Selectivity coefficients values ($\log K^{POT}_{I,A}$) calculated for the ion selective membranes containing 1:1 mixtures of ILs with DOS as plasticisers.

Anion/Type	DBS	Cl	TFMS	DCA	MO	NPOE	MS
ClO_4^-	1.080 ± 0.05	1.473 ± 0.00	1.574 ± 0.08	−0.229 ± 0.01	0.969 ± 0.06	1.223 ± 0.11	0.909 ± 0.08
SCN^-	0.331 ± 0.06	0.564 ± 0.01	0.418 ± 0.01	−1.138 ± 0.01	0.391 ± 0.01	1.293 ± 0.03	−0.142 ± 0.02
NO_3^-	−2.615 ± 0.03	−0.405 ± 0.01	−1.107 ± 0.01	−2.263 ± 0.02	−0.564 ± 0.02	−0.481 ± 0.03	−0.469 ± 0.07
$HCrO_4^-$	−0.547 ± 0.04	−0.426 ± 0.03	−0.495 ± 0.02	−0.613 ± 0.09	−0.384 ± 0.06	−0.180 ± 0.09	−1.102 ± 0.09
OH^-	−2.993 ± 0.07	−3.988 ± 0.03	−5.074 ± 0.32	−5.281 ± 0.01	−2.769 ± 0.02	−5.294 ± 0.11	−3.513 ± 0.03
Br^-	−1.815 ± 0.25	−1.321 ± 0.06	−1.896 ± 0.00	−1.303 ± 0.04	−1.045 ± 0.07	−1.297 ± 0.01	−1.776 ± 0.02
Cl^-	−2.488 ± 0.07	−1.951 ± 0.17	−2.689 ± 0.28	−2.129 ± 0.52	−2.057 ± 0.06	−2.192 ± 0.04	−2.273 ± 0.01
$Fe(CN)_6^{4-}$	−0.551 ± 0.04	−3.159 ± 0.04	−4.240 ± 0.01	−3.514 ± 0.03	−2.995 ± 0.02	−3.670 ± 0.17	−3.560 ± 0.00
SO_4^{2-}	−4.648 ± 0.12	−4.193 ± 0.12	−5.229 ± 0.03	−5.167 ± 0.08	−1.828 ± 0.01	−4.976 ± 0.03	−4.239 ± 0.01

Table S3. Experimental slopes (mV/decade) obtained during potentiometric measurements for ion selective membranes plasticised with ionic liquids and traditional plasticisers at 1:1 ratio.

Anion/Type	DBS	Cl	TFMS	DCA	MO	NPOE	MS
ClO_4^-	−53.5 ± 4.2	−86.8 ± 2.1	−71.0 ± 5.1	−77.7 ± 9.5	−68.6 ± 3.6	−64.2 ± 2.6	−60.6 ± 6.4
SCN^-	−65.1 ± 3.0	−69.0 ± 3.6	−63.3 ± 2.9	−73.0 ± 1.0	−61.4 ± 4.4	−68.8 ± 1.1	−67.2 ± 6.6
I^-	60.2 ± 1.6	61.0 ± 6.7	62.5 ± 3.2	61.5 ± 5.1	57.7 ± 2.9	59.4 ± 3.4	63.6 ± 4.6
NO_3^-	−50.3 ± 5.9	−61.6 ± 9.2	−52.8 ± 3.6	−56.5 ± 1.5	−62.1 ± 3.0	−57.6 ± 2.5	−61.1 ± 3.6
$HCrO_4^-$	54.5 ± 0.4	63.3 ± 2.6	56.0 ± 3.9	55.4 ± 5.3	57.3 ± 4.4	58.6 ± 2.6	60.7 ± 1.3
OH^-	−52.2 ± 2.2	−49.8 ± 1.7	−54.3 ± 4.2	−42.9 ± 1.8	−48.0 ± 17.3	−47.9 ± 3.7	−46.4 ± 1.5
Br^-	57.2 ± 5.3	52.6 ± 3.0	55.1 ± 3.8	53.5 ± 3.1	56.4 ± 5.0	57.3 ± 4.8	60.6 ± 2.6
Cl^-	−54.8 ± 3.6	−59.5 ± 8.2	−54.6 ± 3.0	−55.9 ± 2.0	−56.3 ± 2.8	−55.0 ± 4.0	−56.9 ± 4.2
$Fe(CN)_6^{4-}$	15.2 ± 4.2	16.5 ± 1.9	15.5 ± 1.2	15.4 ± 1.7	12.5 ± 0.5	14.8 ± 2.0	14.9 ± 4.8
SO_4^{2-}	−30.7 ± 1.0	−34.9 ± 2.3	−27.6 ± 1.3	3−4.7 ± 3.4	−33.3 ± 2.2	−31.4 ± 3.6	−33.3 ± 3.8
